# Nonpharmacological interventions for preventing delirium in adult patients with cancer: a systematic review and meta-analysis

**DOI:** 10.1007/s00520-026-10898-2

**Published:** 2026-07-02

**Authors:** Yusuke Kanno, Kaoru Shinsato, Jun Kako, Yoshinobu Matsuda, Shinichiro Inoue, Hitoshi Tanimukai, Saho Wada, Takaaki Hasegawa

**Affiliations:** 1 Graduate School of Health Care Sciences, Institute of Science Tokyo, 1-5-45 Yushima, Bunkyo-Ku, Tokyo, 113-8510 Japan; 2 Department of Psycho-Oncology, Shizuoka Cancer Center, Shizuoka, 411-8777 Japan; 3 Graduate School of Medicine, Mie University, Tsu, 514-8507 Japan; 4 Department of Psychosomatic Internal Medicine, NHO Kinki Chuo Chest Medical Center, Sakai, 591-8555 Japan; 5 Department of Nursing, Niimi University, Niimi, 718-8585 Japan; 6 Graduate School of Nursing, Nagoya City University, Mizuho-Ku, Nagoya, 467-8601 Japan; 7 Division of Quality Assurance Programs, National Cancer Center Institute for Cancer Control, Tokyo, 104-0045 Japan; 8 Center for Psycho-Oncology and Palliative Care, Nagoya City University Hospital, Mizuho-Ku, Nagoya, 467-8601 Japan

**Keywords:** Delirium, Cancer, Nonpharmacological intervention, Multicomponent intervention, Systematic review, Meta-analysis

## Abstract

**Purpose:**

To determine whether nonpharmacological interventions reduce delirium incidence in adult patients with cancer and to identify effective intervention strategies.

**Method:**

We conducted a systematic review and meta-analysis following PRISMA guidelines. PubMed, CENTRAL, CINAHL, and Ichushi-Web were searched through September 30, 2024, for randomized controlled trials (RCTs). Two authors independently extracted data, assessed the risk of bias using the Cochrane Risk of Bias 2 tool, and evaluated the certainty of evidence using the Grading of Recommendations Assessment, Development and Evaluation approach. The primary outcome was delirium incidence. A random-effects model was used for the meta-analysis. The study protocol was registered with UMIN-CTR (No. UMIN000051804; registered August 2, 2023).

**Results:**

Overall, 12 RCTs involving 2,747 patients were included. Interventions were categorized into multicomponent interventions, bright light therapy, rehabilitation, and anesthesia or oxygen management. The meta-analysis of five trials showed that multicomponent interventions significantly reduced delirium incidence (risk ratio [RR], 0.43; 95% confidence interval [CI], 0.31–0.60; moderate-certainty evidence), with benefit predominantly observed in postoperative settings. The evidence for bright light therapy was inconclusive (RR, 0.31; 95%CI, 0.07–1.31; very low-certainty evidence). Most of the included studies had concerns regarding the risk of bias, mainly due to a lack of blinding.

**Conclusion:**

Multicomponent nonpharmacological interventions may reduce delirium incidence in adult patients with cancer, particularly in postoperative or perioperative settings. However, the certainty of evidence is moderate, and the included studies vary in patient populations, intervention components, and healthcare settings. Evidence for standalone interventions and prevention strategies in palliative or end-of-life care remains limited and inconclusive.

**Supplementary Information:**

The online version contains supplementary material available at 10.1007/s00520-026-10898-2.

## Introduction

Delirium is an acute neuropsychiatric syndrome characterized by fluctuating disturbances in attention, awareness, and cognition [1, 2]. It is a common and serious complication in patients who are hospitalized and is associated with adverse outcomes, including prolonged hospitalization, functional and cognitive decline, increased mortality, and substantial distress for patients, families, and healthcare professionals [3, 4]. Delirium is particularly common in patients with cancer, with reported incidence rates of up to 42% in general oncology settings and as high as 88% in the terminal stages of the illness, making it one of the most common mental disorders in this population [5, 6]. The management of established delirium is often challenging, underscoring the critical importance of preventive strategies.

Delirium management strategies are broadly categorized as pharmacological and nonpharmacological. Although pharmacological agents such as antipsychotics are frequently used to manage severe agitation or distress, their efficacy in preventing delirium is limited and associated with significant adverse effects [7, 8]. Accordingly, international clinical practice guidelines have increasingly emphasized prevention and endorsed nonpharmacological interventions as first-line strategies [9–11]. Among patients in general hospital settings, multicomponent interventions targeting modifiable risk factors such as immobility, sensory impairment, dehydration, and sleep disturbance effectively reduce delirium incidence by up to 40% [12, 13]. These interventions are therefore widely accepted as effective preventive strategies in this population.

Despite this evidence, the effectiveness of nonpharmacological delirium prevention strategies in patients with cancer remains insufficiently characterized. To date, most existing systematic reviews and meta-analyses have focused on patients without cancer, such as older medical inpatients, patients in intensive care units, and those undergoing orthopedic or cardiac surgery [10, 12, 14]. This represents a critical evidence gap, because patients with cancer represent a distinct clinical population with unique vulnerabilities, including chemotherapy-related neurotoxicity, cancer-related systemic inflammation, high opioid burden, and paraneoplastic syndromes [15, 16]. Therefore, extrapolating findings from populations without cancer may be inappropriate, leaving clinicians without specific high-quality evidence to guide preventive care for this uniquely vulnerable group. A comprehensive synthesis of the available evidence is therefore required.

Therefore, this systematic review and meta-analysis aimed to evaluate the effectiveness of nonpharmacological interventions in preventing delirium in adult patients with cancer.

## Materials and Methods

### Protocol and registration

This systematic review and meta-analysis was designed, conducted, and reported following the Preferred Reporting Items for Systematic Reviews and Meta-Analyses (PRISMA) 2020 statement [17]. The completed PRISMA 2020 checklist is provided in Supplementary Table 3. The protocol was developed in line with the Minds Manual for Guideline Development 2017 [18] and registered with the University Hospital Medical Information Network Clinical Trials Registry of Japan (registration number: UMIN000051804; registered August 2, 2023).

### Eligibility criteria

Studies were included following the PICOS framework:

- **Participants (P)**: Adult patients (≥ 18 years old) with any type of cancer, irrespective of clinical setting (surgical, medical, or palliative care). Studies with mixed populations were eligible if ≥ 80% of participants had cancer.
- **Interventions (I)**: Any nonpharmacological intervention or program aimed at preventing delirium.
- **Comparators (C)**: Usual care, standard care, or attention control groups.
- **Outcomes (O)**: The primary outcome was delirium incidence, assessed using validated tools (Confusion Assessment Method [CAM] and Nursing Delirium Screening Scale [Nu-DESC]). Secondary outcomes included delirium severity, duration, and length of hospital stay.
- **Study Design (S)**: Randomized controlled trials (RCTs).

Exclusion criteria included nonrandomized studies, studies focusing on delirium treatment (including sedation or causative treatment) rather than prevention, studies involving pediatric populations, and studies not written in English or Japanese.

### Information sources and search strategy

A systematic literature search was performed in PubMed, the Cochrane Central Register of Controlled Trials, CINAHL, and Ichushi-Web (Japan Medical Abstract Society) from inception to August 31, 2023, with an updated search on September 30, 2024. The search strategy combined MeSH terms and keywords related to three concepts—delirium, cancer, and nonpharmacological interventions—in consultation with a medical librarian. Additionally, reference lists of included articles and relevant reviews were manually screened to identify additional studies. The full search strategy for all electronic databases is provided in Supplementary Table 1.

### Study selection

All records retrieved from the database search were imported into Rayyan, a reference management web application (Rayyan Systems Inc., Cambridge, MA, USA), and duplicate records were removed. Subsequently, two authors (Y.K. and K.S.) independently screened the titles and abstracts against the eligibility criteria. Full texts of potentially eligible articles were independently assessed for final inclusion by the same authors. Discrepancies at any stage were resolved through discussion or, if consensus was not reached, through consultation with third authors (J.K., Y.M., and S.W.).

### Data extraction

Two authors (Y.K. and K.S.) independently extracted data from the included studies using a pre-piloted standardized data extraction form. Extracted information included: (1) first author and publication year; (2) participant characteristics (sample size, age, cancer type, and clinical setting); (3) intervention and comparator details; (4) outcome measures, including the tool used to assess delirium; and (5) quantitative data for primary and secondary outcomes. Discrepancies in extracted data were resolved by consensus.

### Risk of bias assessment

Two authors (Y.K. and K.S.) independently assessed the methodological quality of the included RCTs using the revised Cochrane Risk of Bias 2 (RoB 2) tool [19]. This tool assesses potential bias across five distinct domains: (1) randomization, (2) deviations from intended interventions, (3) missing outcome data, (4) outcome measurement, and (5) selective reporting. Each domain was rated as "low risk," "some concerns," or "high risk," yielding an overall risk-of-bias judgment for each study.

### Data synthesis

Meta-analysis was conducted when at least two studies provided sufficient data for the same intervention and outcome. For the primary outcome (delirium incidence), the number of delirium events reported during the intervention period or at discharge was used to calculate pooled risk ratios (RRs) with 95% confidence intervals (CIs). A random-effects model accounted for the expected clinical and methodological heterogeneity between studies. Statistical heterogeneity was quantified using the I^2^ statistic, with values of 25%, 50%, and 75% interpreted as low, moderate, and high heterogeneity, respectively [20]. A narrative synthesis was conducted for interventions and outcomes unsuitable for quantitative pooling, summarizing the findings reported in the original studies. Statistical analyses were performed using EZR (Saitama Medical Center, Jichi Medical University, Saitama, Japan) [21].

### Certainty of evidence assessment

The certainty of evidence for the primary outcome was evaluated using the Grading of Recommendations Assessment, Development and Evaluation (GRADE) approach [22, 23]. Two authors (Y.K. and K.S.) independently rated the evidence as high, moderate, low, or very low based on five domains: risk of bias, inconsistency, indirectness, imprecision, and publication bias. Discrepancies were resolved through discussion. Results were summarized into a Summary of Findings table using the GRADEpro GDT software.

## Results

### Study selection

**Fig. 1:**
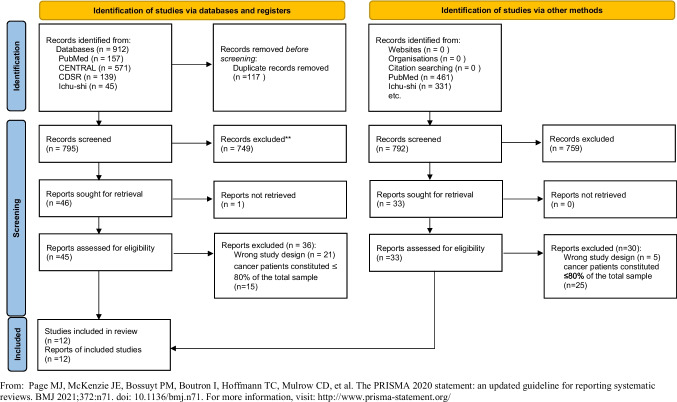
Flowchart for inclusion of studies

The database search initially identified 912 records. After removing 117 duplicates, 795 titles and abstracts were screened, leading to the exclusion of 749 articles. The full texts of the remaining 45 articles were assessed for eligibility, with 36 articles excluded because of ineligible study design or population. An updated search identified three additional articles, resulting in 12 RCTs included in the systematic review and meta-analysis. The selection process is detailed in the PRISMA flow diagram (Fig. 1).

### Characteristics of included studies

The 12 included studies [24–35] are summarized in Table 1. Overall, 2,747 patients were enrolled across these studies, which were published between 2007 and 2024. Most studies were conducted in Asia, including China (*n* = 7) [24, 25, 28, 32–35], Japan (*n* = 2) [30, 31], and Taiwan (*n* = 1) [27], as well as studies from Australia [26] and the Netherlands [29].

**Table 1:** Characteristics of included studies (*n* = 12)

Author (Year, country)	Participants (Analyzed)	Intervention vs Control	Delirium Assessment	Delirium-related outcomes	Other Outcomes
Multi-component interventions					
Chen CY et al. (2024, China)	Non-small cell lung cancer (NSCLC) (n = 140 vs 140)	I: RAM-based CST C: Usual care	Nu-DESC	Incidence: 10.7% (I) vs. 20.7% (C), P = 0.032 Severity: Not reported	Cognitive function (Mini-Cog): Higher in I group at discharge (P = 0.007) Anxiety/Depression (HADS): No significant difference
Wang YY et al. (2020, China)	Older patients (mostly cancer) (n = 152 vs 129)	I: t-HELP C: Usual care	CAM, MDAS	Incidence: 2.6% (I) vs. 19.4% (C); RR 0.14 (95% CI 0.05–0.38) Severity: Severe delirium lower in I group (P = 0.008)	LOS: 12.1 days (I) vs. 16.4 days (C), P < 0.001 Function: Less decline in ADL/IADL in I group (P < 0.001)
Hosei et al. (2020, Australia)	Advanced cancer (Stage IV) (n = 20 vs 20 vs 25)	I: Multicomponent (6 domains, 36 strategies) C: Usual care/Waitlist	Nu-DESC, DRS-R-98	Incidence: 20% (I) vs. 32% (C) vs. 20% (Waitlist), P = 0.5 Severity: No significant difference	Falls: 5% (I) vs. 8% (C) (ns)
Chen CC et al. (2017, Taiwan)	Older abdominal surgery (cancer) (n = 196 vs 179)	I: mHELP (Modified HELP: 3 nurse-led protocols) C: Usual care	CAM	Incidence: 6.6% (I) vs. 15.1% (C), P = 0.008 Severity: Not reported	LOS: Median 12 days (I) vs. 14 days (C), P = 0.04
Guo Y et al. (2016, China)	Older oral cancer (n = 81 vs 79)	I: Multicomponent non-pharmacological interventions C: Usual care	CAM-ICU	Incidence: 15% (I) vs. 31% (C), P = 0.006 Severity: Lower duration in I group (P < 0.001)	Biomarkers: Higher melatonin (P = 0.004) and lower cortisol (P < 0.001) in I group
Hempenius L et al. (2013, The Netherlands)	Frail elderly cancer (n = 127 vs 133)	I: Geriatric liaison intervention (consultation + nurse visits) C: Usual care	DOS, DRS-R-98	Incidence: 9.4% (I) vs. 14.3% (C); OR 0.63 (95% CI 0.29–1.35), ns Severity: No significant difference	LOS: No significant difference Mortality: 7.9% (I) vs. 3.0% (C), ns Complications: No significant difference
Bright light therapy					
Ono et al (2011, Japan)	Esophageal cancer (n = 10 vs 12)	I: Bright light (2 h/morning for 4 days) C: Usual care	NEECHAM	Incidence: 10% (1/10) vs. 41.6% (5/12), ns Severity: No scores below cutoff in I group after Day 4	Activity: Less nighttime movement in I group (P < 0.05) Arrhythmia: Less in I group (P < 0.05)
Taguchi et al (2007, Japan)	Esophageal cancer (n = 6 vs 5)	I: Bright light (5000 lx, 2 h/morning) C: Dim light (600–1000 lx)	NEECHAM	Incidence: Better NEECHAM score in I group on Day 3 (27 vs. 21, P = 0.014)	Ambulation: Started earlier in I group (5.5 vs. 7.6 days), ns
Rehabilitation					
Yuan et al. (2023, China)	Brain tumor (craniotomy) (n = 141 vs 150)	I: FORTITUDE (Early rehab + ADL training) C: Usual care	Nu-DESC	Incidence: 15.6% (I) vs. 28.7% (C), P = 0.007 Duration: Shorter in I group (P < 0.001)	Onset: Delayed in I group (P = 0.035) LOS: Shorter in I group (P = 0.042) ADL: Better in I group at 1 week/1 month
Oxygen Management					
Teng et al. (2024, China)	Thoracic surgery (One-lung ventilation) (n = 60 vs 60)	I: rScO_2-guided lung-protective ventilation C: Standard lung-protective ventilation	CAM	Incidence: 8.5% (I) vs. 23.3% (C), P < 0.05 Severity: Not reported	Biomarkers: Lower S100β(neuron-specific enolase, tumor necrosis factor-α) in I group (P < 0.001) LOS: Shorter in I group (P < 0.001)
Chen J et al (2024, China)	Lower abdominal tumor (Robotic) (n = 51 vs 52)	I: Low PetCO2 (31–40 mmHg) C: High PetCO2 (41–50 mmHg)	CAM	Incidence: 21.6% (I) vs. 15.4% (C), P = 0.419 Severity: No difference	Pain: No difference Breath-holding test: No difference
Lin et al (2021, China)	Gastric/Colorectal cancer (Laparoscopic) (n = 314 vs 316)	I: FiO2 40% C: FiO2 80%	CAM, MDAS	Incidence: 20.38% (I) vs. 18.35% (C), P = 0.402 Severity: MDAS 6.75 vs 7.03, ns	Atelectasis: Lower in 40% FiO2 group (19.1% vs 41.8%, P = 0.026)

*I* Intervention Group, *C* Control Group, *RAM* Roy Adaptation Model, *CST* Cognitive Stimulation Therapy, *HELP* Hospital Elder Life Program, *Nu-DESC* Nursing Delirium Screening Scale, *CAM* Confusion Assessment Method, *MDAS* Memorial Delirium Assessment Scale, *DOS* Delirium Observation Screening Scale, *DRS-R-98* Delirium Rating Scale-Revised-98, *NEECHAM* NEECHAM Confusion Scale, *RASS* Richmond Agitation-Sedation Scale, *LOS* Length of Stay, *ADL* Activities of Daily Living, ns: not significant, *rScO2* Regional cerebral oxygen saturation, *PetCO2* End-tidal carbon dioxide partial pressure, *FiO2* Fraction of inspired oxygen

Interventions were categorized into four groups: multicomponent interventions (*n* = 6) [24–29], bright light therapy (*n* = 2) [30, 31], rehabilitation (*n* = 1) [32], and oxygen management (*n* = 3) [33–35]. The components of these interventions varied across the studies (Table 2). Yet, they commonly included orientation or cognitive stimulation (all studies) and early mobilization (5 studies) [24–27, 29], with sleep enhancement [25, 26, 28, 29] and family involvement [24, 26] in approximately half of the interventions. The majority of studies focused on patients in postoperative or acute care settings [24, 25, 27–35]. Delirium was primarily assessed using the Nu-DESC [24, 26, 32] or the CAM [25, 27, 28, 33–35].

**Table 2:** Components of multi-component interventions in the included studies

Study	Name of Program/Key Concept	Orientation & Cognitive Stimulation	Sleep Enhancement	Early Mobilization & Physical Activity	Vision & Hearing Support	Hydration & Nutrition Management	Family Involvement & Education
Chen CY et al. (2024)	RAM-based CST (Roy Adaptation Model-based Cognitive Stimulation Therapy)	X		X			X
Wang YY et al. (2020)	t-HELP (Tailored, family-involved Hospital Elder Life Program)	X	X	X	X	X	X
Hosei et al. (2020)	PRESERVE Pilot (Multicomponent non-pharmacological intervention)	X	X	X	X	X	X
Chen CC et al. (2017)	mHELP (Modified Hospital Elder Life Program)	X		X		X	
Guo Y et al. (2016)	MNI (Multicomponent, Nonpharmacological Interventions)	X	X		X	X	
Hempenius L et al. (2013)	Geriatric Liaison Intervention (Consultation & Nurse visits)	X	X	X	X	X	

**RAM-based CST**: Focuses on physiology (exercise), self-concept (memory games), role function (daily living skills), and interdependence (family support) modes

**t-HELP**: A tailored version of HELP involving family members to deliver protocols (orientation, meal assistance, mobility, sleep, etc.)

**PRESERVE**: Targeted 6 domains: Eating/drinking, Sleep, Exercise, Reorientation, Vision/Hearing, and Family partnership

**mHELP**: Simplified to 3 core protocols administered by a nurse: orienting communication, oral/nutritional assistance, and early mobilization

**MNI**: Included psychological support, orientation (clocks/calendars), sensory aids, sleep cycle maintenance (light/noise control), music, and early feeding

**Geriatric Liaison**: Involved preoperative assessment and daily nurse visits checking a 9-item list including orientation, mobility, senses, sleep, and intake

### Risk of bias assessment

The methodological quality of the 12 included RCTs was assessed using the RoB 2 tool, with results summarized in Supplementary Table 2 and Supplementary Fig. 1. Overall, five studies had a low risk of bias. Six were classified as having some concerns, primarily due to deviations from the intended interventions (domain 2). This was largely attributed to the lack of blinding, which is often inherent in nonpharmacological trials. One study was judged to have a high risk of bias due to issues in the randomization process (domain 1) and a high rate of missing outcome data (domain 3).

### Synthesis of results

#### Primary outcome: incidence of delirium

##### Multicomponent interventions

**Fig. 2:**
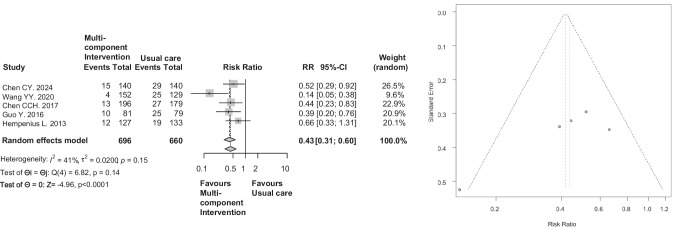
Forest plot and corresponding funnel plot of the effect of multicomponent interventions on the incidence of delirium

Overall, six RCTs evaluated multicomponent interventions. A total of five studies [24, 25, 27–29] included patients in postoperative or general hospital settings, while one [26] involved patients with advanced cancer in a palliative care unit. The meta-analysis of the five hospital-based studies (n = 1,356) [24, 25, 27–29] showed that multicomponent interventions significantly reduced delirium incidence compared with usual care (RR, 0.43; 95% CI, 0.31–0.60; p < 0.00001; moderate-certainty evidence), with moderate heterogeneity (I^2^ = 41%) (Fig. 2). In contrast, the palliative care study [26] reported no significant differences between intervention and control groups. Due to the distinct patient populations and settings, this study was analyzed separately.

##### Bright light therapy

**Fig. 3:**
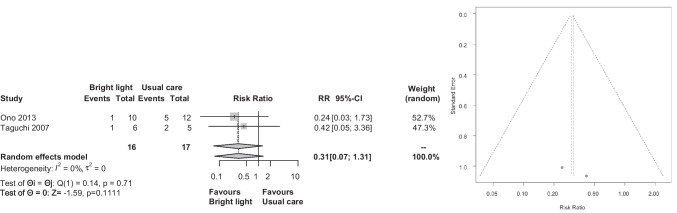
Forest plot and corresponding funnel plot of the effect of bright light therapy on the incidence of delirium

Two RCTs [30, 31] involving 33 patients evaluated the effects of bright light therapy. The meta-analysis showed no statistically significant difference in delirium incidence between the intervention and control groups (RR, 0.31; 95% CI, 0.07–1.31; p = 0.1111), with no observed statistical heterogeneity (I^2^ = 0%). However, evidence certainty was very low due to serious imprecision, rendering the findings inconclusive (Fig. 3).

##### Narrative synthesis of other interventions

A meta-analysis was not performed for rehabilitation and oxygen management due to the limited number of studies and substantial clinical heterogeneity. One RCT [32] of early rehabilitation in patients with brain tumors reported a significantly lower delirium incidence in the intervention group (15.6%) compared with the usual care group (28.7%). The three studies [33–35] on oxygen management used different surgical protocols, precluding quantitative pooling.

### Secondary outcomes

A meta-analysis was not conducted for secondary outcomes (i.e., length of hospital stay, delirium duration, and delirium severity) due to the limited number of studies and the use of different measurement scales. Consequently, these findings were synthesized narratively. Specifically, shorter hospital stays were reported in four studies [25, 27, 32, 33]. A shorter delirium duration was noted in several studies across different intervention types. Furthermore, two studies [25, 32] reported improved patient function at discharge, measured as Activities of Daily Living scores. A single study [24] observed improved postoperative cognitive function and higher patient satisfaction. A detailed summary of all secondary outcomes is presented in Table 1.

### Certainty of evidence

Based on the GRADE assessment, the evidence for multicomponent interventions reducing delirium was moderate, downgraded due to risk of bias from lack of blinding. In contrast, the evidence for bright light therapy was very low, due to the risk of bias and serious imprecision resulting from the small sample size and wide CIs crossing the line of no effect. A detailed certainty assessment is presented in the Summary of Findings (Table 3).

**Table 3:** Certainty of evidence (GRADE) for the effect of multi-component interventions and bright light therapy on the incidence of delirium

Non-pharmacological interventions compared to usual care for adult patients with cancer						
Patient or population: Adult patients with cancer						
Setting: Hospital						
Intervention: Non-pharmacological interventions (e.g., multicomponent interventions, bright light therapy)						
Comparison: Usual care						
Outcome	*Anticipated absolute effects (95% CI)		Relative effect (95% CI)	№ of participants (studies)	Certainty of the evidence (GRADE)	Comments
	Risk with Usual care	Risk with Intervention				
Multicomponent interventions						
Delirium Incidence	189 per 1,000	81 per 1,000 (114 to 59)	RR 0.43 (0.31 to 0.60)	1356 (5 RCT)	⨁⨁⨁◯ MODERATE	Downgraded one level for risk of bias (lack of blinding), as approximately half of the included studies were assessed as having "some concerns"
Bright light therapy						
Delirium Incidence	412 per 1,000	128 per 1,000 (539 to 29)	RR 0.31 (0.07 to 1.31)	33 (2 RCT)	⨁◯◯◯ VERY LOW	Downgraded one level for risk of bias (lack of blinding) and two levels for very serious imprecision due to the extremely small sample size (n = 33) and wide confidence intervals crossing the line of no effect

^*^The risk in the intervention group (and its 95% confidence interval) is based on the assumed risk in the comparison group and the relative effect of the intervention (and its 95% CI)

CI: Confidence interval; RR: Risk ratio

**GRADE Working Group grades of evidence**

**High certainty:** we are very confident that the true effect lies close to that of the estimate of the effect

**Moderate certainty:** we are moderately confident in the effect estimate: the true effect is likely to be close to the estimate of the effect, but there is a possibility that it is substantially different. **Low certainty:** our confidence in the effect estimate is limited: the true effect may be substantially different from the estimate of the effect

**Very low certainty:** we have very little confidence in the effect estimate: the true effect is likely to be substantially different from the estimate of effect

## Discussion

To our knowledge, this is the first systematic review and meta-analysis to exclusively evaluate nonpharmacological interventions for delirium prevention in adult patients with cancer. The principal finding of this review was that multicomponent interventions were associated with a lower incidence of delirium among patients with cancer in postoperative or perioperative settings. The certainty of this evidence was moderate according to the GRADE approach, indicating that the true effect was likely to be close to the estimate but may have differed because of limitations in the primary studies, including risk of bias and clinical heterogeneity. However, the effectiveness of these interventions in patients with advanced cancer in palliative or end-of-life care settings remains unclear. Current evidence for standalone interventions, such as bright light therapy, rehabilitation, and oxygen therapy, is insufficient to draw definitive conclusions. Specifically, the certainty of evidence for bright light therapy was very low due to serious limitations. While these findings are promising, they must be interpreted cautiously because of methodological concerns, particularly the risk of bias in the included studies.

The pooled estimate suggests a relative reduction in delirium incidence of approximately 57% among postoperative or perioperative patients with cancer. Although this effect estimate is clinically important, it should be interpreted in the context of moderate-certainty evidence, some concerns regarding risk of bias, and variation in intervention components and study populations. This result is consistent with prior meta-analyses in surgical patients without cancer and supports the importance of proactively addressing multiple modifiable risk factors (e.g., immobility, dehydration, and sleep disturbance) during the perioperative period [14, 36]. Importantly, the term "multicomponent intervention" covered a broad range of programs rather than a single standardized package. Across the included trials, all programs incorporated orientation or cognitive stimulation, whereas early mobilization, sleep enhancement, hydration or nutrition support, sensory support, family involvement, and staff education were included inconsistently. Therefore, the observed pooled effect should be interpreted as reflecting structured multicomponent prevention programs as a broad intervention class, rather than evidence for any single component or fixed protocol. The current evidence does not allow determining specific components, combinations, intensity, or delivery models essential for delirium prevention in oncology settings.

Evidence for multicomponent interventions in palliative or end-of-life care remains limited. Only one small feasibility trial conducted in a palliative care unit was identified, and it did not demonstrate a significant reduction in delirium incidence. Accordingly, differences between postoperative and palliative settings should be interpreted cautiously and not considered definitive evidence of differential effectiveness. Nonetheless, delirium prevention may require distinct approaches across the cancer trajectory, because terminal-phase delirium is often associated with complex and potentially irreversible etiologies, including multiorgan failure, systemic inflammation, medication burden, and direct or indirect central nervous system involvement [37–40]. Adequately powered studies are needed before conclusions can be drawn regarding prevention strategies in palliative care settings.

Regarding standalone interventions, our analysis did not identify a significant benefit of bright light therapy. This finding aligns with our GRADE assessment, which rated the certainty of evidence as very low. However, these results must be interpreted with caution. Although the biological rationale for light therapy in regulating circadian rhythms remains compelling [41, 42], the evidence was downgraded for very serious imprecision due to the extremely small sample size (*n* = 33). Notably, no significant heterogeneity was observed (I^2^ = 0%), suggesting a consistent direction of effect across the included studies despite their limited size. Therefore, our findings do not refute the potential of light therapy but rather highlight that the current evidence base is inconclusive, and a Type II error cannot be excluded. Although individual RCTs evaluating rehabilitation and oxygen management showed promising results, the scarcity of data precluded a meta-analysis. Overall, this review systematically maps the existing evidence landscape, distinguishing interventions with more established efficacy from those requiring further investigation.

### Implications for clinical practice and research

Our findings have implications for clinical practice. The current evidence may inform the development and implementation of structured, interdisciplinary delirium prevention programs for patients with cancer in the postoperative or perioperative setting. However, implementing these resource-intensive interventions requires significant commitment from nursing staff and multidisciplinary teams, and their transferability to different healthcare systems should be considered carefully. In palliative or end-of-life care settings, clinicians should recognize that the evidence for delirium prevention is currently limited. Nevertheless, even when multicomponent interventions do not significantly reduce delirium incidence, meeting fundamental care needs—such as orientation and comfort—remains essential for maintaining patient dignity, particularly as priorities shift towards symptom management and family communication. From a utilitarian perspective, balancing the potential clinical benefits of delirium prevention with the costs and workload on healthcare providers is a critical ethical challenge. Future studies should include cost-effectiveness analyses to evaluate the feasibility of intensive interventions in routine oncology practices.

From a research perspective, this systematic review highlights several critical avenues for future research. First, a major evidence gap exists regarding delirium prevention in nonsurgical and advanced populations with cancer. High-quality RCTs are needed to develop and evaluate interventions tailored for these patients, potentially focusing on different targets than those used in postoperative settings. Second, further well-designed RCTs with standardized protocols are required to clarify the effectiveness of standalone interventions, such as bright light therapy and rehabilitation. Finally, the heterogeneity in multicomponent interventions and outcome measures across studies presents a significant challenge. Establishing a consensus on a core outcome set for delirium research in oncology would substantially advance the field by improving comparability across trials and enabling more robust and informative meta-analyses.

### Limitations

This study has some limitations that must be acknowledged. First, the methodological quality of the primary studies is a key constraint. As reflected in the GRADE assessment, approximately half of the included RCTs were judged to raise "some concerns" regarding risk of bias, primarily due to the inherent difficulty of blinding personnel and participants in nonpharmacological trials. This may have introduced a performance or detection bias, potentially leading to a modest overestimation of the true intervention effects.

Second, the included cancer populations were clinically heterogeneous. Although most studies were conducted in postoperative or acute care settings, the broader review question included patients across different cancer trajectories, including medical, surgical, and palliative care contexts. Delirium in these settings may differ in its underlying mechanisms, precipitating factors, reversibility, and responsiveness to preventive interventions. Therefore, the findings should not be generalized to all patients with cancer, particularly those receiving palliative or end-of-life care. In addition, although we categorized the interventions, significant clinical heterogeneity remained, particularly within the "multicomponent" category. As shown in Table 2, the specific intervention components (e.g., sleep enhancement, family involvement), delivery models, and intensity varied across studies. Although statistical heterogeneity was moderate (I^2^ = 41%), further quantitative exploration of heterogeneity was not feasible because of the small number of studies included in the meta-analysis. Potential sources of heterogeneity include cancer type, surgical procedure, baseline delirium risk, age and frailty, intervention intensity, degree of family involvement, staff training, timing and duration of intervention delivery, and delirium assessment tools. These factors may have influenced both baseline delirium incidence and the apparent effectiveness of the interventions.

Third, the study population may have involved potential indirectness. We included trials with mixed populations when at least 80% of participants had cancer, which was a pragmatic decision to avoid excluding otherwise relevant oncology evidence. However, this criterion means that a minority of participants in some studies may not have had cancer. Therefore, the findings should be interpreted as evidence primarily applicable to populations composed predominantly of patients with cancer, rather than as purely cancer-specific evidence.

Fourth, the generalizability of the findings may have also been limited by the geographical distribution of the included studies. Most trials were conducted in Asia, particularly China, Japan, and Taiwan, with fewer studies from Europe or other regions. Differences in perioperative care pathways, nurse staffing, family involvement in care, hospital length of stay, delirium screening practices, and availability of geriatric or palliative care services may affect the transferability of these interventions to other healthcare systems.

Fifth, most of the included studies relied on screening tools (e.g., Nu-DESC and CAM) rather than the gold-standard diagnostic criteria (e.g., Diagnostic and Statistical Manual of Mental Disorders, Fifth Edition) to identify delirium. Differences in sensitivity and specificity across these instruments may have influenced the estimated incidence rates and the apparent effectiveness of preventive effects.

## Conclusion

This systematic review and meta-analysis suggest that multicomponent nonpharmacological interventions may reduce delirium incidence in adult patients with cancer, with the clearest evidence currently available in postoperative or perioperative settings. However, the certainty of evidence is moderate, and interpretation is limited by the risk of bias, clinical heterogeneity, variation in intervention components, potential population indirectness, and the predominance of studies from Asian healthcare settings. Evidence remains insufficient for standalone interventions and for prevention strategies in palliative or end-of-life care. Further high-quality, adequately powered trials are needed to identify which intervention components are most effective and how delirium prevention should be adapted across different stages of cancer care.

## Supplementary Information

Below is the link to the electronic supplementary material.

Supplementary file1 (DOCX 4.16 MB)

## Data Availability

All relevant data have been included in this article.
