# Supplementary figures and images for: Nonpharmacological interventions for preventing delirium in adult patients with cancer: a systematic review and meta-analysis
Source: Support Care Cancer. 2026 Jul 2;34(7):720. doi: 10.1007/s00520-026-10898-2 (PMC13328200; doi:10.1007/s00520-026-10898-2)

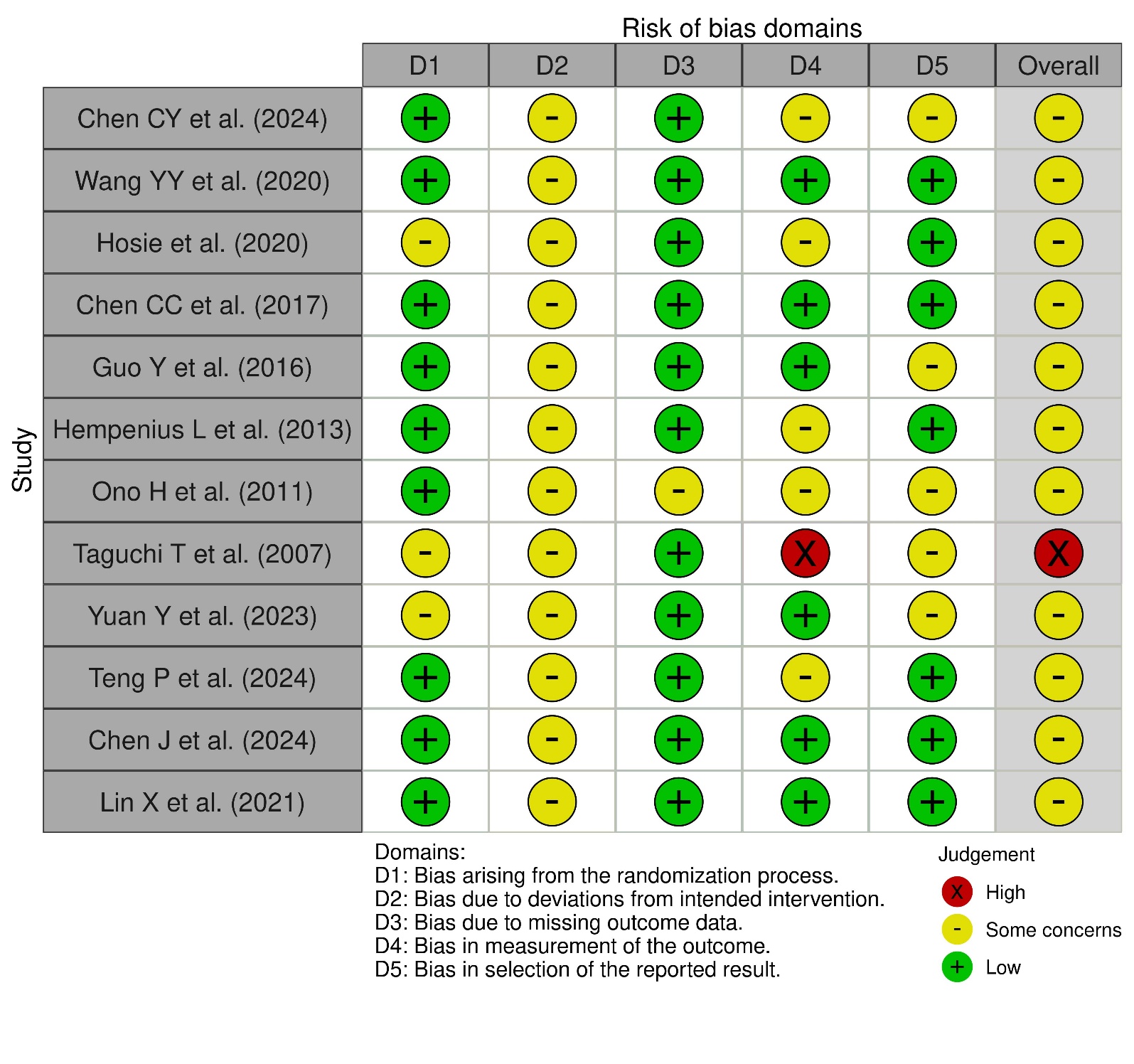


Supplementary Figure 1. Risk of bias assessment for inclusion of studies

Supplement: Supplementary file 1 — Supplementary file1 (DOCX 4.16 MB) [file 520_2026_10898_MOESM1_ESM.docx]
